# Severe polycystic liver diseases: hepatectomy or waiting for liver transplantation? Two case reports

**DOI:** 10.1097/MD.0000000000018176

**Published:** 2019-12-10

**Authors:** Zeyu Zhang, Kuan Hu, Jiajin Yang, Yufan Zhou, Zhiming Wang, Yun Huang

**Affiliations:** Department of Hepatobiliary Surgery, Xiangya Hospital, Central South University, Changsha, Hunan, China.

**Keywords:** hepatectomy, liver parenchyma, polycystic liver disease, subradical polycystic hepatectomy, treatment

## Abstract

**Introduction:**

Choice of treatment in patients with symptomatic polycystic liver diseases (PLD) remains controversial. Various surgical procedures aiming at eliminating symptomatic cysts are widely used in mild and advanced PLD patients, but liver transplantation is currently recommended as the only curative treatment especially in severe cases.

**Patient concerns:**

Case 1: A 51-year-old male was admitted for severe eating disorder and dyspnea for 2 months. He had been diagnosed as PLD, PKD, and hypertension for 9 years, with only antihypertensive drug therapy. No significant family history could be traced.

Case 2: A 54-year-old female was admitted to our hospital for ventosity during nearly 5 years. She had been diagnosed as PLD and hypertension for 22 years, for which only aspiration-sclerotherapy therapy was performed for multiple times. Both her mother and sister were diagnosed with PLD previously.

**Diagnosis:**

They were diagnosed as PLD by medical history, family history, and computed tomography scan (multiple cysts dispersively presenting in the liver).

**Interventions:**

The 2 patients underwent hepatectomy with fenestration, and were well recovered with no mortality.

**Outcomes:**

While case 1 only experienced relief of symptoms, case 2 experienced massive growth of hepatic parenchyma, which indicated positive prognosis and showed the possibility to avoid or at least postpone liver transplantation for a long time, considering the lack of liver parenchyma is one of the main reason for urgency of liver transplantation.

**Conclusion:**

Here we described subradical polycystic hepatectomy, a special form of hepatectomy with fenestration modified by us, as a safe and effective treatment to potentially achieve long-term effects in PLD patients.

## 1 Introduction

Polycystic liver disease (PLD) is known for cholangiopathy involving abnormal protein expression in cilia, presenting as multiple cysts spreading dispersively in liver lobes.^[1]^ PLD mostly exists in 2 different situations, as the main manifestation of isolated polycystic liver disease (PCLD) or a secondary manifestation of autosomal dominant polycystic kidney disease (ADPKD).^[2]^ PLD usually progresses with an increase of number and volume of liver cysts and eventually causes severe hepatomegaly followed by insufferable symptoms, such as abdominal pain, abdominal distension, dyspepsia, early satiety, dyspnea, and back pain.^[3]^

The treatments of PLD are limited, including surgical options such as aspiration-sclerotherapy, fenestration, hepatectomy, liver transplantation, and medical options such as somatostatin analogs and renin-angiotensin-aldosterone system.^[4]^ However, the efficacy of these treatments remains uncertain except liver transplantation which is now the only curative treatment of PLD.^[5]^

As a treatment of PLD, hepatectomy is usually performed under the circumstance that the patient obtains significant symptoms with spared liver segment and acceptable liver function.^[6,7]^ In the past years, hepatectomy is commonly considered not to be the first-line treatment on account of its high morbidity and mortality, as well as the possibility to affect future liver transplantation.^[2,8,9]^ Recently, some studies showed that hepatectomy could significantly relieve symptoms and reach long-term control of PLD,^[10,11]^ However, the efficacy of hepatectomy is still controversial in severe PLD patients.^[3,12]^

## 2 Case presentation

### 2.1 Case 1

**Figure 1:**
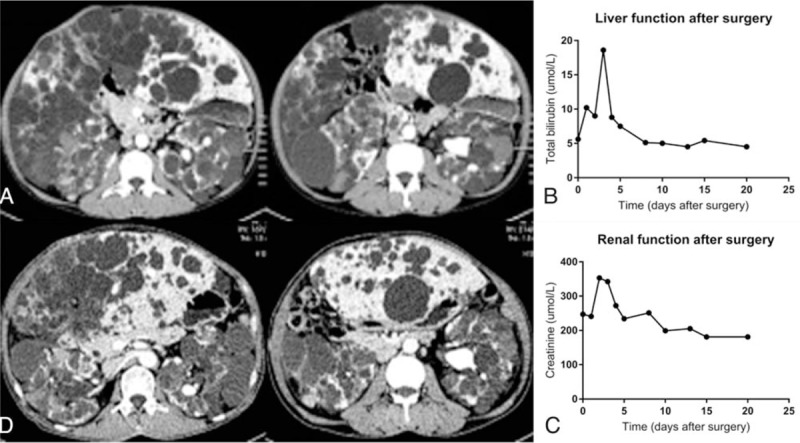
(A) Computed tomographic (CT) scan of the abdomen evidencing enlarged liver and kidneys with multiple cysts and there was few liver parenchyma. (B) Liver function was fully recovered after surgery. (C) Renal function was significantly improved after surgery. (D) Postoperative CT scan of the abdomen showed the growth of liver parenchyma was not as fast as expected (13.8%). CT = computed tomography.

A 51-year-old male was admitted for severe eating disorder and dyspnea for 2 months. He had been diagnosed as PLD, PKD, and hypertension for 9 years, with only antihypertensive drug therapy. No significant family history could be traced. On admission, his blood pressure was 125/89 mm Hg with performance statu (PS) score = 2. Coagulation function tests and liver function tests were all within normal limits, while hematology tests and renal function tests showed mild anemia and renal impairment (hemoglobin 121 g/L, blood urea nitrogen 19.29 mmol/L [2.60–7.50 mmol/L normally], creatinine 247.0 μmol/L [41.0–111.0 μmol/L normally]). CA 19–9 was up to 219.84 KU/L (<35.00 KU/L normally). Abdominal computed tomography (CT) scan showed large number of cysts diffusely distributed all over the liver (Fig. 1A). Portal veins and hepatic veins were well shown in CT scan without sign of embolism, however, being narrowed and displaced due to multiple and huge cysts. The kidneys were enlarged with multiple cysts and calculus. After comprehensive assessment, instead of performing regular hepatectomy aiming at reducing liver volume, subradical polycystic hepatectomy was planned. Subradical polycystic hepatectomy was a special form of hepatectomy with fenestration modified by us, where all the cyst-dominated hepatic segments were removed with cyst fenestration performed among unresected cysts. According to measurement of hepatic segments volume and remnant liver volume through three-dimensional reconstruction and surgery planning system, left lateral lobe of liver was supposed to be conserved during the surgery (617 mL, residual liver volume/standard liver volume [RLV/SLV]: 54.5%). Unfortunately, a blood loss of 3000 mL was met when the right lobe of liver was removed and we had to stop to prevent further blood loss. Cyst fenestration was performed for those unresectable cysts. Somatostatin was used after the surgery and the patient was favorably recovered. Mean volume of drainage of ascites was 1500 mL per day. Creatinine continuously decreased and reached 181 μmol/L when discharging (Fig. 1 B and C). After 80 days of follow-up, huge improvement of life quality (PS score = 0) was achieved except occasionally slight ventosity during exercises and post cibum. Unfortunately, three-dimensional reconstruction showed that his hepatic parenchyma only grew by 13.8% (85 mL) contrasting to remnant liver volume in surgery planning system (Fig. 1D).

### 2.2 Case 2

**Figure 2:**
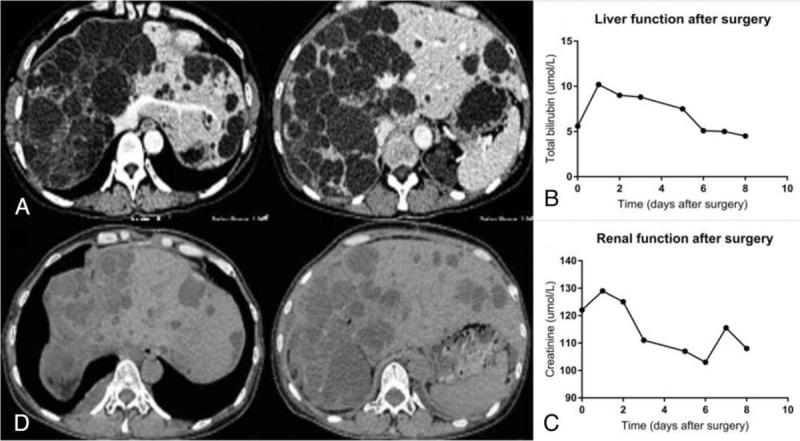
(A) Computed tomographic (CT) scan of the abdomen evidencing enlarged liver with multiple cysts. (B) Liver function was fully recovered after surgery. (C) Renal function was improved after surgery and became normal when discharge. (D) Postoperative CT scan of the abdomen. Enhancement CT was replaced by normal CT when following up because of allergy. CT = computed tomography.

A 54-year-old female was admitted for ventosity during nearly 5 years. She had been diagnosed as PLD, PKD, and hypertension for 22 years, for which only aspiration-sclerotherapy therapy was performed for multiple times. Both her mother and sister were diagnosed as PLD previously. On admission, her blood pressure was 161/104 mm Hg with PS score = 2. Coagulation function test was within normal limits, but hematology test, liver function test, and renal function test showed anemia, malnutrition, and renal dysfunction, respectively (hemoglobin 109 g/L, albumin 37.5 g/L, blood urea nitrogen 6.06 mmol/L, creatinine 120.0 μmol/L). CA 19–9 was normal (27.03 KU/L). CT scan of abdomen showed large numbers of cysts diffusely distributed over the liver, especially right lobe and left medial lobe, while left lateral liver was barely involved (Fig. 2A). Portal veins and hepatic veins were well showed in CT scan without sign of embolism. The kidneys were enlarged with multiple cysts. After comprehensive assessment, subradical polycystic hepatectomy was planned. According to measurement of hepatic segments volume and remnant liver volume through three-dimensional reconstruction and surgery planning system, most of left lateral lobe of liver (709 mL, RLV/SLV: 68.0%) was supposed to be conserved. The surgery went smoothly and the right lobe and left internal lobe dominated by cysts were removed. Cyst fenestration was performed for remnant cysts. The electrotome and bipolar electrocoagulation were used to destroy cyst wall for reducing production of ascites. Somatostatin was used after surgery and the patient was favorably recovered. Blood pressure maintained normal without any drugs since the third day after surgery, and mean volume of drainage of ascites was 200 mL per day. Creatinine continuously decreased and reached normal limit (108.0 μmol/L) before discharging (Fig. 2 B and C). After 70 days of follow-up, three-dimensional reconstruction showed that her hepatic parenchyma grew by 40.3% (286 mL) contrasting to remnant liver volume in surgery plan (Fig. 2D). Moreover, her life quality greatly improved (PS score = 0) without any symptom and her blood pressure stayed normal without any drug.

## 3 Discussion

Adult PLD usually occurs as an extrarenal manifestation of ADPKD with a prevalence of 1:2000 to 1:5000, or less commonly, as a main manifestation in PCLD.^[13]^ The diagnosis of PLD is made when more than 20 cysts are presenting in liver. With PLD being an autosomal dominant genetic disease, the number and size of hepatic cysts in PLD patients increase along with age. Abdominal pain, fatigue, and dyspnea are the most common initial complaints in symptomatic PLD. As far as treatment for PLD is concerned, different surgical procedures including aspiration-sclerotherapy, fenestration, hepatectomy, transcatheter arterial embolization, and liver transplantation are used in symptomatic PLD.^[3]^ Among them, liver transplantation offers the only curative option and is recommended to severe PLD patients, but it is a costly and risky surgical procedure with the lifelong intake of immunosuppressant drug. Moreover, lacking of donor and low allocating priority of PLD make it difficult for PLD patient to get liver transplantation.^[14]^ It makes us to reconsider: is there an alternative way?

Here we reported 2 cases of adult PLD that experienced severe hepatomegaly-related symptoms and underwent hepatectomy and fenestration. According to Clavien–Dindo classification, postoperative complications including bile leakage, controlled low-grade ascites, hemorrhage, or drug allergy (case 1 grade II, and case 2 grade I) were observed. Both patients were well recovered with no mortality. Moreover, the relief of symptoms was instantaneous and remarkable during followed-up. According to Gigot classification of PLD,^[15]^ case 1 and 2 were classified as Gigot type III. While there is no doubt that liver transplantation is strongly recommend for Gigot type III patients,^[3,5,15]^ in our experience reported above, it seemed Gigot III type was not the absolute contraindication of hepatectomy with fenestration. What is more, in the current treatments of PLD, the therapeutic intention of either fenestration or hepatic resection for PLD is to achieve relief of symptoms, which can be achieved by reducing liver volume.^[15,16]^ But If we look at the longer run, can we pursue long-term effects through hepatectomy?

Subradical polycystic hepatectomy was a modified surgery by us as a special form of hepatectomy with fenestration in PLD patients, which was introduced previously. Comparing with regular hepatectomy in PLD, this procedure emphasized opinion that all the cyst-dominated hepatic segments (in another word nonfunctional segments), instead of only part of them for purpose of reducing liver volume, should be removed to not only alleviate symptoms, but also prompt the growth of liver parenchyma. In this report, unexpected minor hepatic parenchyma growth was observed in case 1 (13.8%). We considered the failure of subradical polycystic hepatectomy was responsible for the low parenchyma growth in case 1, and subradical polycystic hepatectomy had advantages in prompting growth of liver parenchyma, and thus achieving long-term effects comparing to regular surgery.

Liver dysfunction, which is one of the main reasons leading to the urge of transplantation, is essentially the insufficiency of hepatic parenchyma. Meanwhile, liver is unique in its strong ability of regeneration in response to injury such as hepatectomy and chemical liver injuries.^[17]^ We considered the growth of hepatic parenchyma might play a pivotal role in PLD treatment. Although hepatectomy can not completely stop the progression of PLD, but if the volume of hepatic parenchyma is increasing, we can greatly slow down the disease progression and prolong the time of transplantation. In our report, massive growth of hepatic parenchyma (41.0%) was observed in case 2. From our perspective, this was due to the increased energy per unit liver volume and altered hemodynamic factors, which resembled the concept in associating liver partition and portal vein ligation for staged hepatectomy.^[18]^ On the other hand, hepatectomy itself may also play a role in prompting the growth of hepatic parenchyma by making extra space in abdominal cavity. Anyway, subradical polycystic hepatectomy achieved these by maximumly removing nonfunctional liver segments.

However, the volume of hepatic parenchyma will continuously decrease and distorted blood vessel will be hard to protect while cysts are growing. It will be gradually difficult to achieve subradical polycystic hepatectomy along with progressing of PLD. Should this procedure be performed as soon as possible?

The majority of PLD patients will not require treatment, and there is no need to perform any treatment in asymptomatic patients.^[2]^ We considered the indication of subradical polycystic hepatectomy was basically the same as regular hepatectomy with fenestration, which should be performed in patients with severe symptoms. Besides, subradical polycystic hepatectomy may indicate a larger extent of resection comparing with regular hepatectomy, so we only recommended it when patient condition was acceptable and sufficient volume of hepatic parenchyma and portal vein and hepatic vein of preserved segments were ensured. Furthermore, considering the possible relationship between timing of surgery and postoperative growth of liver parenchyma, subsequent studies will be performed toward this issue.

Hypertension and renal dysfunction are the most common comorbidities in PKD with PLD. Hypertension occurs in 60% of patients before renal impairment in PKD, and is universal in chronic kidney disease stages 4 to 5.^[19]^ Activation of the renin-angiotensin-aldosterone system, vascular dysfunction, related to ciliopathy and other factors have all been found to be involved in the development of hypertension in ADPKD.^[20]^ Amelioration of hypertension was observed in case 2 after surgery without handling renal cysts. However, the long-term effects and mechanism should be determined in future researches. Moreover, ADPKD is characterized by increasing kidney size and gradually decreasing renal function. The majority of patients require dialysis by the age of 60.^[21]^ The average age of end-stage renal disease (ESRD) in ADPKD patients has not improved, despite improved overall survival owing to a higher prevalence of renal transplantation and better ESRD care.^[22]^ In our 2 cases, creatinine significantly decreased after surgery even without any renal treatment. We supposed it could be the decreased intra-abdominal pressure that affected renal function. However, lacking of intra-abdominal pressure data made it hard to prove the hypothesis, so future studies would be needed to go further.

It can be quite a challenge when it comes to the choice of treatment for PLD patients. In our experience, severe symptomatic patients with advanced hepatomegaly may benefit from hepatectomy instead of waiting for transplantation. The hepatectomy must be thorough that will be followed by massive growth of hepatic parenchyma which is essential for slowing progression of PLD down as well as improving prognosis of patient with PLD. Thus, subradical polycystic hepatectomy, a special form of hepatectomy with fenestration, could be a safe and effective treatment to potentially achieve long-term effects in PLD patients.

## Author contributions

**Investigation:** Zeyu Zhang.

**Resources:** Zeyu Zhang.

**Supervision:** Zhiming Wang, Yun Huang.

**Writing – original draft:** Zeyu Zhang.

**Writing – review & editing:** Zeyu Zhang, Kuan Hu, Jiajin Yang, Yufan Zhou, Yun Huang.
